# Career interest data trends in era information technology of high school students at Surabaya, Indonesia

**DOI:** 10.1016/j.dib.2020.105480

**Published:** 2020-04-18

**Authors:** Mudhar, F. Danardana Murwani, Imanuel Hitipeuw, Hetti Rahmawati

**Affiliations:** a Faculty of Psychological Education, Universitas Negeri Malang, Indonesia; b Faculty of Economics, Universitas Negeri Malang, Indonesia; c Faculty of Psychological Education, Universitas Negeri Malang, Indonesia

**Keywords:** Career interest, Trends, Holland theory, RIASEC

## Abstract

This research was conducted to determine trends in the career interests of high school students in Surabaya, East Java, Indonesia. The sample size was 981 consisting of 488 men and 493 women. The instrument used was a career interest scale that was compiled based on Holland's theory with six RIASEC domains (Realistic, Investigative, Artistic, Social, Enterprising, and Conventional). The study design uses non-experimental, data collection through questionnaires given directly. Data were analyzed descriptively without using an explicit theoretical model. The career fields that are in high demand by high school students are the conventional fields that reach 42.30%, while the less desirable areas are the investigative fields which are only 3.98%. There are differences in career interests between men and women. Men prefer more realistic, artistic and enterprising fields, while women prefer social and conventional fields.

Specifications Table

**Table:** 

Subject	Applied Psychology
Specific subject area	Vocational Psychology, “Career Interest”
Type of data	Table and Figure
How data were acquired	This data was obtained from the results of the assessment using a career interest scale of 981 high school students in class XII in Surabaya.
Data format	Raw and Analyzed
Parameters for data collection	Career Interest Data is collected using a career interest scale based on RIASEC theory from Holland.
Description of data collection	Students answer statements with a choice of strongly disagree, disagree, doubt, agree and strongly agree, scores range from 1, 2, 3, 4 and 5. Scores are added up and averaged to determine the highest career interest.
Data source location	High School Students (SMA) City: Surabaya, Jawa Timur Country: Indonesia Surabaya geographically is at 07˚09′00 “- 07˚21′00” South Latitude and 112˚3′- 112˚54′ East Longitude, and with google maps: https://www.google.co.id/maps/@-7.2564168,112.7509655,15z
Data accessibility	The data available in Mendeley Data: http://dx.doi.org/10.17632/wsh9c9xxg8.11

## Value of the data

• Data can be used to see differences in career interests in the context of cultural differences.
• These data can be used to investigate possible changes in career interest trends as a result of the development of science and technology.
• This data is useful for providing information to policy makers in this case the ministry of education and culture (kemendikbud) in order to provide schools that are in line with students’ career interests.
• Data can be used by teachers, especially guidance and counseling teachers, to develop students’ career interests.
• Data can be used for scientists interested in longitudinal studies of changes in vocational behavior and career interests.

## 1 Data description

Data were collected using a career interest scale compiled based on Holland's theory, which distinguishes individuals into six scopes or domains of interest, namely Realistic, Investigative, Artistic, Social, Enterprising, and Conventional [1]. Realistic domain consists of 7 items, Investigative domain comprises 7 items, there are 7 items in Artistic domain, 6 items in Social domain, 8 items for Enterprising domain, and 4 items in Conventional domain (Table 1, Table 2, Table 3, Table 4, Table 5, Table 6).

**Table 1:** Items measuring Realistic.

No.	Items	Mean	SD	Loading Factor	Cronbach's Alpha	CR	AVE
R-1	I like repairing power tools	2.43	1.161	0.842	0.881	0.958	0.764
R-2	I am able to repair electronic goods	2.51	1.088	0.856			
R-4	I want to be a technician	2.39	1.084	0.924			
R-9	I can fix household furniture	2.82	1.222	0.923			
R-11	I want to be an electrician	3.30	1.034	0.930			
R-20	I want to be a mechanical / mechanical expert	2.72	1.353	0.801			
R-39	I like fixing mechanical tools/machine	2.73	1.384	0.833

**Table 2:** Items measuring Investigative.

No.	Items	Mean	SD	Loading Factor	Cronbach's Alpha	CR	AVE
I-6	I enjoy doing activities in the laboratory	2.99	1.148	0.894	0.824	0.963	0.789
I-7	I am able to understand chemical formulas	2.35	1.083	0.840			
I-10	I want to be a biologist	2.66	1.127	0.901			
I-18	I want to be a chemist	2.27	1.055	0.862			
I-19	I enjoy reading scientific books or magazines	3.00	1.122	0.911			
I-21	I want to be a researcher	2.99	1.139	0.908			
I-22	I am happy to take part in the scientific writing competition	2.45	0.981	0.900

**Table 3:** Items measuring Artistic.

No.	Items	Mean	SD	Loading Factor	Cronbach's Alpha	CR	AVE
A-12	I love to sketch, draw, or paint	3.15	1.23	0.816	0.793	0.949	0.726
A-13	I love to appear in art performances	3.05	1.20	0.881			
A-14	I enjoy attending art performances or exhibitions	3.42	1.11	0.865			
A-17	I am able to write stories / poems	3.06	1.11	0.911			
A-28	I want to be an Artist	2.93	1.18	0.829			
A-31	I can play a musical instrument	3.01	1.13	0.836			
A-38	I want to be a musician	2.93	1.18	0.823

**Table 4:** Items measuring Social.

No.	Items	Mean	SD	Loading Factor	Cronbach's Alpha	CR	AVE
S-15	I am able to teach children	3.44	1.01	0.887	0.584	0.929	0.689
S-23	I enjoy participating in social activities	3.85	1.01	0.916			
S-24	I'm happy to help people who are struggling	4.45	0.74	0.708			
S-27	I want to be a teacher	3.08	1.23	0.810			
S-33	I want to be a counselor	2.69	1.04	0.851			
S-36	I am able to help a friend who is having problems	4.22	0.80	0.790

**Table 5:** Items measuring Enterprising.

No.	Items	Mean	SD	Loading Factor	Cronbach's Alpha	CR	AVE
E-5	I want to be a Distributor	3.02	1.06	0.841	0.652	0.933	0.637
E-8	I want to be an Businessman	4.33	0.91	0.665			
E-16	I was once elected as an organization administrator	3.22	1.32	0.861			
E-25	I once led the organization	2.82	1.30	0.809			
E-29	I am happy to discuss political matters	2.86	1.17	0.846			
E-30	I love being the leader of an organization	2.97	1.17	0.846			
E-32	I am able to argue	3.13	1.12	0.780			
E-34	I want to be an Entrepreneur	3.39	1.08	0.714

**Table 6:** Items measuring Conventional.

No.	Items	Mean	SD	Loading Factor	Cronbach's Alpha	CR	AVE
C-3	I am able to arrange documents neatly	3.88	0.85	0.816	0.429	0.868	0.624
C-26	I can save documents well	3.77	0.92	0.825			
C-35	I like to organize / save important documents	3.84	0.94	0.822			
C-37	I want to be a bank employee	3.48	1.71	0.687			

## 2 Experimental design, materials, and methods

Data collected through non-experimental surveys, researchers collect all data from respondents through questionnaires given directly. The data is descriptive survey data designed to determine the career interests of high school students in the city of Surabaya, East Java, Indonesia. The use of this descriptive design without using an explicit theoretical model [2], besides the descriptive survey design is more efficient and more effective in investigating the description of career interests that are most preferred by students.

### 2.1 Participant

The population in this survey is class XII high school students in the city of Surabaya, East Java, Indonesia in the academic year 2019. The sampling technique includes non-random sampling, the determination of the sample is based on the division of the area or region [3], South Surabaya, Central Surabaya, and North Surabaya (http://dx.doi.org/10.17632/wsh9c9xxg8.11). The researcher went directly to the high school which had been previously selected as a place of research. This research involved 6 high schools in the city of Surabaya. Researchers gave questionnaires directly to students. The data collection process of this research took place from December 2, 2019, to December 10, 2019. Participants were 981 students of class XII high school students in the city of Surabaya, 49.75% were male participants and 50.25% were female.

### 2.2 Measures and data analysis

The measurement uses a Career Interest Scale with 39 items divided into 6 scopes (Realistic, Investigative, Artistic, Social, Enterprising, and Conventional). All items were scored on a 5-point scale, ranging from 1 (strongly disagree) to 5 (strongly agree) Likert types of scale [4]. The sum and average of the scoring of each individual in each interest scope or domain are then calculated. The career interests of each student are determined from the highest average score (see data http://dx.doi.org/10.17632/wsh9c9xxg8.11).

**Fig. 1:**
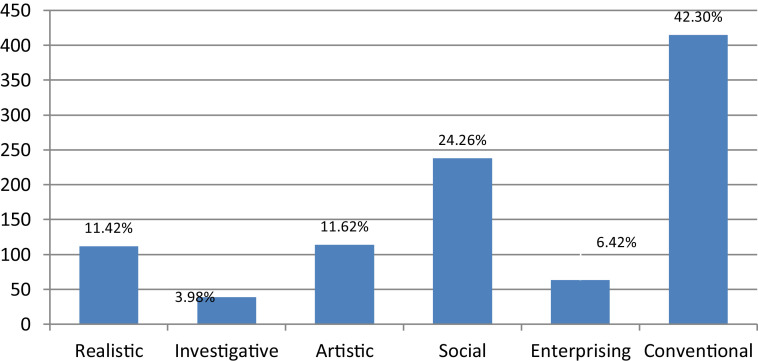
Career interest trends in 2019.

**Fig. 2:**
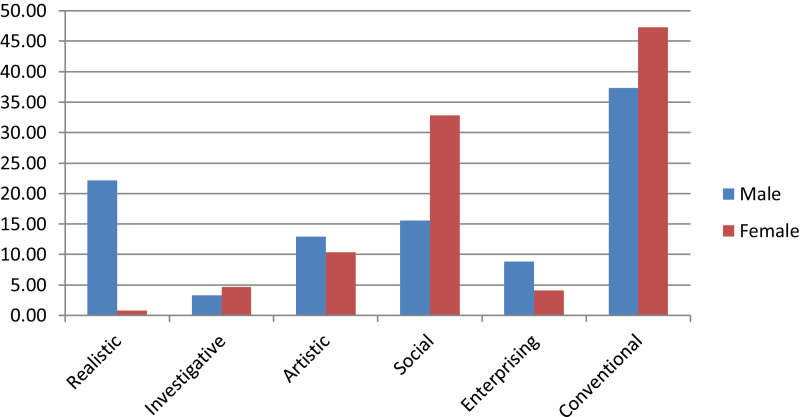
Comparison of career interests between male and female.

Career interests of students are determined by the highest average score in each category or area of career interest. The career fields that are in high demand by high school students are the conventional fields which reach 42.30% (Fig. 1). Whereas the less desirable or less interested sectors were investigative fields which were only 3.98% (Fig. 1). When comparing career interests between men and women (Table 7 and Fig. 2) it appears that there are differences in the fields of career interest between men and women. The scope of career interests favored by men is in the realistic, artistic and enterprising fields, while women prefer the social and conventional fields. In the field of investigative less interested by both men and women.

**Table 7:** Distribution of Career Interests.

Interest Scope	Sex			
	Male (*n* = 488)		Female (*n* = 493)	
Realistic	108	22,13%	4	0,81%
Investigative	16	3,28%	23	4,67%
Artistic	63	12,91%	51	10,34%
Social	76	15,57%	162	32,86%
Enterprising	43	8,81%	20	4,06%
Conventional	182	37,30%	233	47,26%
Total	488	100%	493	100%
